# Tuberous Sclerosis Complex: New Insights into Pathogenesis and Therapeutic Breakthroughs

**DOI:** 10.3390/life15030368

**Published:** 2025-02-26

**Authors:** Aurora Alexandra Jurca, Alexandru Daniel Jurca, Codruta Diana Petchesi, Dan Bembea, Claudia Maria Jurca, Emilia Severin, Sanziana Jurca, Cosmin Mihai Vesa

**Affiliations:** 1 Doctoral School of Biological and Biomedical Sciences, University of Oradea, 410087 Oradea, Romania; jurca.auroraalexandra@student.uoradea.ro; 2 Department of Preclinical Disciplines, Faculty of Medicine and Pharmacy, University of Oradea, 1 December Sq., 410081 Oradea, Romania; petchesidiana@uoradea.ro (C.D.P.); danbembea@yahoo.com (D.B.); claudiajurca@uoradea.ro (C.M.J.); cosmin.vesa@csud.uoradea.ro (C.M.V.); 3 Regional Center of Medical Genetics Bihor, County Emergency Clinical Hospital Oradea (Part of ERN THACA), 410469 Oradea, Romania; 4 Department of Genetics, University of Medicine and Pharmacy “Carol Davila”, Dionisie Lupu Street, Number 37, District 2, 020021 Bucharest, Romania; 5 Faculty of Medicine and Pharmacy, University of Oradea, December Sq., 410081 Oradea, Romania; jurca.sanzianaiulia@student.uoradea.ro

**Keywords:** TSC1 gene, TSC2 gene, hamartomas, mTOR inhibitors

## Abstract

**Background/Objectives**: Tuberous sclerosis complex (TSC) is a rare, autosomal dominant genetic disorder caused by mutations in the *TSC1* and *TSC2* genes, which disrupt the regulation of the mammalian target of rapamycin (mTOR) pathway, a critical regulator of cellular growth. The disorder presents as a multisystem condition, with benign tumors (hamartomas) developing in organs such as the brain, skin, heart, kidneys, and lungs, leading to significant clinical variability and impact on quality of life. This review aims to summarize recent advances in the understanding of TSC pathogenesis and clinical variability and evaluate the therapeutic breakthroughs in targeted treatments. **Methods**: A narrative review was conducted using various available databases. We applied objective evaluation metrics, such as the impact factor of the journals and the citation count, to assess the quality of the studies. **Results**: Targeted therapies, particularly mTOR inhibitors (mTORis), have shown efficacy in reducing hamartoma size, improving neuropsychiatric symptoms, and enhancing patient outcomes. Despite these advances, variability in disease expression poses challenges in diagnosis and individualized management strategies. **Conclusions**: Challenges such as early diagnosis, optimizing long-term outcomes, and addressing residual unmet needs remain critical. Future research should prioritize precision medicine approaches and patient-centered care models within centers of expertise to improve treatment efficacy and quality of life for individuals with TSC.

## 1. Introduction

Tuberous sclerosis complex (TSC) is a rare genetic disorder with autosomal dominant inheritance and multisystem involvement. It is a genetic condition with complete penetrance but variable expressivity. The clinical presentation is associated with severe morbidity and potential mortality, making early diagnosis, lifelong surveillance, and appropriate management crucial. Alongside neurofibromatosis types 1 and 2 (NF1 and NF2), Sturge–Weber syndrome (SWS), von Hippel–Lindau disease (VHL), Incontinentia pigmenti (IP), hereditary hemorrhagic telangiectasia (HHT), ataxia–telangiectasia (A-T), and basal cell nevus syndrome (BCNS), TSC is part of the group of neurocutaneous syndromes. This broad group includes both classical phacomatoses (NF, SWS, VHL) and related conditions (IP, HHT, A-T, BCNS). The clinical presentation is characterized by the simultaneous involvement of the nervous system and skin (a shared embryonic origin: neuroectoderm) and a predisposition to tumor development in various organs, particularly the brain, spinal cord, and skin [1].

The concept of TSC was introduced 160 years ago. The first observations were made in 1835 when Pierre François Olive Rayer, a renowned French dermatologist, published a colored illustration in his dermatological works depicting a patient with facial lesions that appear to correspond to angiofibromas, one of the cutaneous manifestations of tuberous sclerosis. Later, in 1862, von Recklinghausen described cases of cardiac myxomas and cerebral sclerosis in a newborn who died shortly after birth. However, the most significant contribution came from the French neurologist Désiré-Magloire Bourneville, who, in 1880, described and named “tuberous sclerosis” after identifying neuropathological changes in a young patient with seizures, hemiplegia, intellectual disability, and renal tumors [2].

The clinical manifestations of TSC are varied, depending on the age of onset, the extent of systemic involvement, and severity. Consequently, clinical features may present dynamically throughout an individual’s life [3]. The key characteristics of TSC include the presence of hamartomatous tumors in multiple organ systems (brain, skin, heart, kidneys, eyes, etc.), seizures, and neuropsychiatric manifestations such as behavioral disorders, autism, and cognitive impairments. A hallmark of TSC is its pronounced phenotypic and genotypic heterogeneity. The underlying pathology is attributed to pathogenic variants in two critical genes: TSC1, located on chromosome 9, and TSC2, located on chromosome 16. These genes encode the proteins hamartin (TSC1) and tuberin (TSC2), which play pivotal roles in regulating cell growth and proliferation by acting as inhibitors of the mechanistic target of rapamycin (mTOR) signaling pathway [3].

Tuberous sclerosis complex (TSC) has an incidence ranging from 1 in 6000 to 1 in 10,000 live births. In Europe, the prevalence is estimated to range between 1 in 11,500 and 1 in 25,000. Current global estimates for TSC typically range from 800,000 to 1.3 million individuals worldwide, which include diagnosed and undiagnosed cases (due to the variability in phenotypic expression, severity, and age of onset). These estimates are continually updated as more data become available through better diagnostic technologies, genetic screenings, and larger epidemiological studies. The condition shows no predilection for sex or gender or ethnicity [4,5,6].

Living with TSC poses numerous challenges for patients and their families, as the disorder often requires ongoing multidisciplinary care to manage its complex clinical manifestations and the associated psychosocial impacts. Lifelong surveillance is necessary to monitor organ involvement and prevent complications, underscoring the importance of integrated healthcare approaches.

In recent years, the establishment of European Reference Networks (ERNs) has significantly improved the management of and research into rare diseases like TSC. ERNs are collaborative networks that connect healthcare providers across Europe, facilitating multidisciplinary approaches to diagnosis and treatment. The ERN for Rare Neurological Disorders (ERN-RND) includes TSC and promotes the sharing of best practices, knowledge, and innovative research initiatives among clinicians and researchers. This collaboration aims to enhance patient outcomes by improving access to specialized care and advancing the understanding of rare conditions.

In addition, the TOSCA project (TuberOus SClerosis Registry to increase disease Awareness) has emerged as a vital initiative dedicated to enhancing patient care and research in TSC. This international, multicenter registry collects comprehensive data on patients, including their clinical characteristics, treatment responses, and long-term health outcomes. By standardizing data collection across diverse patient populations, the TOSCA project aims to identify unmet needs, inform clinical guidelines, and promote the development of targeted therapies [7].

The treatment of tuberous sclerosis complex (TSC) is both pathogenetic and symptomatic. The introduction of mTOR inhibitors (mTORis), such as sirolimus and everolimus, has provided new insights into the management of the tumor-related forms of TSC, significantly improving patient quality of life by controlling tumor growth and reducing systemic complications. While these therapies represent a major advancement, ongoing research seeks to address unmet clinical needs and explore novel therapeutic options, including the potential of gene therapy.

This narrative review aims to provide a comprehensive update on the available data regarding tuberous sclerosis, focusing on the underlying pathogenic mechanisms, clinical progression, and emerging therapeutic strategies, based on the latest scientific advancements. The search was limited to English-language articles published between 2019 and 2024, with key studies published prior to 2019 included for context.

## 2. Clinical Features

The clinical presentation of tuberous sclerosis complex (TSC) is highly variable, primarily due to the development of benign tumor masses, most commonly affecting the skin, brain, eyes, lungs, kidneys, and heart. However, any part of the body can potentially be involved. The severity of symptoms varies based on the size and location of the lesions, along with the unique characteristics of each patient [4].

The diagnostic criteria for tuberous sclerosis complex (TSC), initially established at the 2012 Consensus Conference, were reviewed by each working group, which subsequently recommended retaining, modifying, adding, or removing certain major or minor criteria. The updated clinical diagnostic criteria, revised in 2021 through the Tuberous Sclerosis Complex International Consensus Conference, include only two changes compared to the previous version and now consist of 11 major features and 7 minor features [8]. In the context of tuberous sclerosis complex (TSC), the diagnostic criteria have undergone significant refinement over time, reflecting a deeper understanding of the condition. The inclusion of features such as “multiple cortical tubers and/or radial migration lines” highlights the distinct and specific brain abnormalities associated with TSC. These criteria play an important role in improving diagnostic accuracy, particularly through the use of advanced neuroimaging techniques like MRI, which reveal these characteristic findings [9].

Historically, the term “cortical dysplasias” was used more broadly to describe various brain abnormalities. However, this lacked the specificity required for identifying TSC with confidence. The updated criteria now focus on the unique neuroanatomical features of TSC, such as cortical tubers—disorganized regions of the brain with altered cellular architecture—and radial migration lines, which reflect disrupted neuronal migration during development. This shift enhances diagnostic precision, ensuring that individuals with subtle or atypical presentations of TSC are more accurately identified.

The evolution of these criteria underscores the importance of integrating clinical, imaging, and genetic data for a comprehensive approach to diagnosing TSC, ultimately aiding in timely intervention and management.

Table 1 presents the criteria for the clinical diagnosis of tuberous sclerosis complex (TSC), based on the major and minor disease criteria established by the international TSC consortium.

### 2.1. Cutaneous Involvement

Skin manifestations, observed in 90% of patients with TSC, hold significant diagnostic value, as they often raise initial suspicion of the disease. The most characteristic skin lesions include facial angiofibromas, hypomelanotic macules (some resembling the shape of an ash leaf, or ash-leaf spots), the shagreen patch (most commonly located in the lumbosacral region), and ungual fibromas. These lesions can be disfiguring or cause discomfort. Longitudinal follow-up using digital photography can aid in tracking changes in skin lesions, while dermatological evaluation by an experienced specialist, with the use of a Wood’s lamp, is particularly valuable for detecting hypomelanotic macules. Sun protection is recommended for both adults and children due to the photosensitivity of hypomelanotic macules and the association of ultraviolet (UV) radiation with mutations observed in angiofibromas. Annual dermatological evaluations are advised for children with TSC, while for adults, the frequency of evaluation depends on the severity of the skin lesions. Any rapid change in the size or number of skin lesions, as well as lesions causing pain or bleeding, should be promptly evaluated and treated. Monitoring skin lesions with serial photographs is recommended to facilitate accurate assessment and timely intervention [9]. For extensive or disfiguring lesions, as well as those prone to bleeding or those causing pain, targeted intervention with mTOR pathway inhibitors, ablative lasers, or surgical excision is indicated [10,11,12].

### 2.2. Renal Involvement

The two most common renal manifestations in tuberous sclerosis complex (TSC) are angiomyolipomas (AMLs), observed in 70–80% of affected individuals, and renal cysts, found in 10–20%. These lesions can lead to complications such as chronic kidney disease (CKD) or secondary hypertension, and regular monitoring is essential. Abdominal MRI is the preferred imaging modality for detecting these lesions across all age groups, while abdominal CT with contrast can be useful for identifying lipid-poor angiomyolipomas and renal cysts [13,14].

Large AMLs (>4 cm) pose a significant risk of spontaneous hemorrhage, requiring careful surveillance. In cases of symptomatic or rapidly growing AMLs, treatment with mTOR inhibitors such as everolimus has shown to be effective in reducing lesion size and mitigating associated risks. Additionally, embolization or nephron-sparing surgery may be indicated for ruptured AMLs.

Blood pressure monitoring is crucial due to the increased risk of secondary hypertension in patients with TSC [15]. Abdominal MRI may also detect other findings associated with TSC, such as aortic aneurysms, liver hamartomas, and neuroendocrine tumors in abdominal organs.

At diagnosis, renal function should be assessed using glomerular filtration rate (GFR) calculations in both adults and children. For individuals with reduced muscle mass, where creatinine-based GFR estimates may be inaccurate, serum cystatin C levels provide a more reliable assessment [16]. Annual evaluations of renal function, proteinuria, and blood pressure are recommended for patients with normal findings, with more frequent assessments for those with renal insufficiency or hypertension.

Lipid-poor angiomyolipomas, a common complication in pediatric patients with TSC, are characterized by their slow growth (<5 mm/year) and typical intrarenal growth pattern. Unlike renal cell carcinoma, which compresses surrounding tissues, AMLs grow within the kidney. As 25–30% of lipid-poor AMLs may be missed on ultrasound, MRI is the preferred imaging technique. If differentiation from renal cell carcinoma is necessary, a biopsy with specific antibody staining, such as HMB-45, can confirm the diagnosis [16,17].

Patients with the TSC2-PKD1 contiguous gene deletion are at higher risk for severe renal manifestations, including early-onset polycystic kidney disease. Regular screening and tailored management strategies are particularly important in this subgroup.

### 2.3. Pulmonary Involvement

Pulmonary involvement is less common in tuberous sclerosis complex (TSC) but can lead to significant morbidity in affected individuals. The most frequent pulmonary condition associated with TSC is lymphangioleiomyomatosis (LAM), which occurs in up to 40% of adult females with TSC and rarely in men [18]. LAM is characterized by the infiltration of lung tissue by abnormal smooth muscle cells, resulting in the formation of cystic lesions that can lead to complications such as pneumothorax, pleural effusion, and hemoptysis [19,20].

Another pulmonary condition associated with TSC is Multifocal Micronodular Pneumocyte Hyperplasia (MMPH), a benign proliferation of type II pneumocytes. MMPH manifests as small (2–14 mm), ground-glass nodules in imaging and is typically asymptomatic. These nodules are rarely associated with LAM and do not require biopsy unless malignancy is suspected [21,22].

For pulmonary evaluation in TSC, chest CT is recommended for symptomatic patients aged 18 years or older, regardless of sex. Ultra-low-radiation-dose protocols and advanced imaging techniques, such as minimum-intensity projection, can enhance the detection of small cysts while minimizing radiation exposure [23,24]. Pulmonary function tests, including spirometry, are advised at baseline and then annually in asymptomatic patients. More frequent testing (every 3–6 months) is reserved for those with newly diagnosed LAM, worsening symptoms, or advanced lung disease, and to monitor the response to mTOR inhibitor (mTORi) therapy.

The key elements of a medical history include a family history of lung disease, occupational or environmental exposures (e.g., tobacco use), and symptoms such as exertional dyspnea, chronic cough, hemoptysis, chest pain, or prior episodes of pneumothorax.

Although not yet standard in clinical practice, the biomarker vascular endothelial growth factor-D (VEGF-D) has demonstrated utility in diagnosing LAM in women with TSC. Elevated VEGF-D levels are highly specific to LAM and can help differentiate it from other cystic lung diseases [25].

### 2.4. Cardiac Involvement

Cardiac involvement in tuberous sclerosis complex (TSC) most commonly presents as cardiac rhabdomyomas, benign tumors of the myocardium. Approximately 47 to 70% of people with TSC have cardiac rhabdomyomas. These tumors are frequently detected in infancy or early childhood and are associated with complications such as heart failure, arrhythmias, and, in severe cases, outflow obstruction. However, rhabdomyomas typically regress spontaneously after infancy, reducing the need for intervention in most cases.

At the time of diagnosis, all individuals with TSC should undergo an age-appropriate cardiac evaluation [26]. For pediatric patients, particularly those under the age of three, an echocardiogram and 12-lead electrocardiogram (EKG) are essential to detect rhabdomyomas and assess for cardiac arrhythmias. For patients identified prenatally with cardiac rhabdomyomas on ultrasound, fetal echocardiography can provide critical information about the risk of heart failure after birth.

In adults, routine echocardiography is not recommended unless there are cardiac symptoms or a history of rhabdomyomas requiring follow-up. Instead, an EKG is advised, as conduction abnormalities such as atrioventricular block or Wolff–Parkinson–White syndrome may be present and can influence medication choices or therapeutic dosages [27].

Patients with a history of arrhythmias or abnormal ECG findings should be cautioned about the potential arrhythmic risks of certain medications, including those that prolong the QT interval. A cardiology consultation is essential before initiating any new medication or supplement in these patients [9].

For symptomatic patients or those with additional risk factors identified on initial screening, ongoing evaluations in a specialized cardiology clinic may be necessary. Advanced imaging techniques, such as cardiac MRI, can provide further characterization of rhabdomyomas and other cardiac anomalies when echocardiography results are inconclusive.

### 2.5. Ophthalmologic Involvement

Ocular manifestations are common in tuberous sclerosis complex (TSC), as the eye shares its embryologic origin (ectoderm) with the nervous system and skin. Retinal astrocytic hamartomas are the most frequent ocular finding, present in 30% to 50% of patients with TSC, and are bilateral in approximately 43% of cases and multiple in about 40%. These lesions are typically located in the posterior pole, near the vascular arcades, or adjacent to the optic nerve. While they are generally benign and asymptomatic, complications such as optic nerve compression, exudation, or hemorrhage can rarely occur [9].

Another common finding is retinal achromic patches, which are depigmented areas observed in up to 39% of cases [28]. Although their clinical significance is limited, they can aid in the diagnosis of TSC.

Routine ophthalmologic evaluation, including fundoscopy, is recommended at the time of diagnosis to identify retinal astrocytic hamartomas and retinal achromic patches. For individuals with ocular involvement, annual follow-ups are advised to monitor for changes or complications.

Treatment for retinal astrocytic hamartomas is typically reserved for cases with significant complications, such as optic nerve compression, exudation, or bleeding. Options include laser intervention, photodynamic therapy, intravitreal anti-VEGF agents, intravitreal steroids, or surgical excision. Recently, mTOR inhibitors (mTORis) have shown promise in managing symptomatic or problematic hamartomas [29,30].

### 2.6. Gastrointestinal Involvement

Gastrointestinal manifestations in tuberous sclerosis complex (TSC) are relatively uncommon and not considered hallmark features of the disease. Occasionally, benign gastrointestinal polyps or other nonspecific gastrointestinal lesions may be observed, but their clinical significance in TSC is limited. Routine gastrointestinal evaluation at the time of diagnosis is not recommended unless the patient presents with specific symptoms (e.g., abdominal pain, bleeding, or changes in bowel habits) or has a relevant medical history that warrants further investigation [9].

### 2.7. Dental Evaluation

During dental evaluations, intraoral fibromas and enamel defects are commonly detected. Approximately 70% of adults with TSC develop gingival fibromas which may cause irritation and may affect tooth alignment. Enamel pits are observed in 90–100% of permanent teeth but are rarely seen in deciduous teeth [9].

For infants diagnosed with tuberous sclerosis complex (TSC), a dental consultation is recommended at the eruption of the first tooth and ideally before the age of 12 months. This should be accompanied by registration at a dental clinic for regular follow-up. If multiple lesions are found, additional screening for other TSC-related lesions may be considered [3,12,31].

For patients who experience difficulties with oral hygiene, more frequent dental visits, such as every 3 months, are advised to ensure proper care and monitoring [32]. Panoramic X-rays (OPG) are recommended to evaluate tooth eruption, identify any abnormalities, and assess delayed eruption. However, due to the radiation exposure from OPG imaging, it is important to use the lowest possible radiation dose, particularly in young children, to minimize risks. Ultra-low-radiation protocols or alternative imaging techniques, such as bitewing radiographs, should be considered when appropriate. To prevent periodontal issues, sealants or fluoride treatments may be used, and any intraoral fibromas that interfere with oral function or cause discomfort should be surgically excised.

### 2.8. Neurological and Psychiatric Aspects

Approximately 80% and possibly a higher percentage of people with TSC have brain involvement, but this does not always have debilitating effects. Epileptic seizures are the most common and significant symptoms in tuberous sclerosis complex (TSC). They can profoundly affect patients’ quality of life and are often associated with various neurological and psychosocial consequences. Seizures can vary widely in severity and frequency, ranging from isolated episodes to recurrent seizures or even status epilepticus, a medical emergency characterized by continuous or repeated seizures [33]. The frequency of seizures in TSC is variable and differs from patient to patient. Some individuals may experience seizures early in life, while others may develop them later, and some may never experience seizures. Seizure types commonly seen in TSC include generalized tonic–clonic seizures and complex partial seizures (with or without secondary generalization), as well as absence seizures and myoclonic seizures [34,35].

Subependymal giant cell astrocytoma (SEGA) is a serious complication in TSC that often requires urgent intervention. Patients with SEGA who experience acute deterioration from obstructive hydrocephalus or tumor hemorrhage may need emergency surgery, with advanced techniques improving safety in appropriately selected cases. While endoscopic resections can be performed for smaller lesions, their use is limited. Laser interstitial thermal therapy (LITT) has been reported in case studies but lacks long-term outcome data. Early intervention by experienced medical teams is essential. For larger tumors associated with hydrocephalus, temporary external ventricular drainage or ventriculoperitoneal shunting may be necessary. Neoadjuvant therapy with mTOR inhibitors has shown promise in improving surgical outcomes by reducing tumor size, enhancing the tumor–brain interface, and decreasing vascularization [36,37].

**Figure 1:**
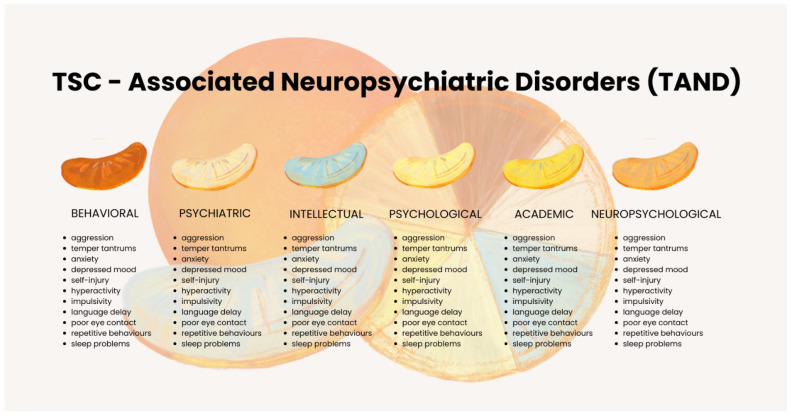
The spectrum of neuropsychiatric disorders associated with tuberous sclerosis complex (TSC) (TAND) adapted from the TAND Consortium [40].

In addition to seizures, patients with TSC may experience various neurological complications, including cognitive impairments, learning disabilities, autism, and behavioral disorders. The term TAND (tuberous sclerosis complex-associated neuropsychiatric disorders) was introduced in 2013 to describe the interrelated neuropsychiatric manifestations frequently observed in TSC. These include challenges in behavioral, psychiatric, intellectual, academic, neuropsychological, and psychosocial domains. TAND-related issues are prevalent and often have the greatest impact on individuals with TSC, but they may not be adequately addressed or managed by current treatments. To assist in identifying these challenges, the Lifetime TAND Checklist (TAND-L), developed in 2015, serves as a screening tool for individuals of all ages. It aims to guide discussions between patients, caregivers, and healthcare providers, helping to identify and manage neuropsychiatric concerns in TSC (Figure 1) [38,39].

Annual screening for TAND should be performed throughout the patient’s lifetime using validated tools, such as the TAND Checklist, or more frequently if clinically indicated. If concerns arise during screening, individuals should be referred to appropriate specialists for the evaluation, diagnosis, and management of the relevant TAND manifestations. Formal TAND evaluations are also recommended at key developmental milestones. Many individuals with TSC face academic or school-related challenges, and they may benefit from an individualized education plan (IEP). These difficulties can significantly affect occupational functioning during childhood and adulthood [39]. TAND evaluation is essential at diagnosis to address any identified issues. Parents or caregivers of dependent adults should be educated about potential urgent TAND manifestations. Children with TSC should initially be referred to a pediatric neurologist with experience in TSC-related epilepsy. Similarly, adults should be evaluated by an adult neurologist familiar with TSC-related epilepsy. Ongoing management can be coordinated by a general pediatrician or neurologist, as needed [41].

Regardless of the presence or absence of seizures or epileptic spasms, an initial and routine electroencephalogram (EEG) is recommended to assess both awake and REM sleep states. Psychological and social support for family members is also useful in helping them adapt to TSC and TAND diagnosis. Therefore, healthcare strategies should be in place to support caregivers’ well-being, and areas requiring immediate or early intervention should be promptly identified. Patients should be referred, on a case-by-case basis, to specialized centers with expertise in evaluating and treating TSC-related conditions to initiate appropriate therapies [13].

## 3. Paraclinical and Imaging Diagnosis

Paraclinical and imaging investigations are also involved in the diagnosis of TSC. The choice of investigation depends on the affected organ or system, and the clinician should decide based on clinical suspicion.

### 3.1. Central Nervous System (CNS) Involvement

If the brain is suspected to be involved, an MRI of the brain is typically the first choice. This helps to spot signs like cortical and subcortical tubers, subependymal nodules, and subependymal giant cell astrocytomas (SEGAs), which are common in TSC. If an MRI is not available, a CT scan or even a transfontanellar ultrasound (for babies) can provide useful information, though they may not detect all the details an MRI can show [42,43].

### 3.2. Cardiac Involvement

For individuals with suspected cardiac rhabdomyomas, an echocardiogram is usually the go-to test. In some cases, an MRI might be used for a clearer, more detailed picture of the heart.

### 3.3. Renal Involvement

When it comes to the kidneys, renal ultrasound is commonly used to look for angiomyolipomas and cysts, which are seen in about 80% of people with TSC. If needed, abdominal CT or abdominal MRI may give a more detailed view of the kidneys.

### 3.4. Pulmonary Involvement

If pulmonary lymphangioleiomyomatosis (LAM) is suspected, a high-resolution CT scan of the chest is often the best way to visualize the lungs and assess any abnormalities.

### 3.5. Ophthalmic Involvement

For the detection of retinal hamartomas and other eye issues, a fundoscopy and slit-lamp examination are essential. These exams are especially important since eye involvement can often go unnoticed but can be a key part of the diagnosis.

### 3.6. Dental Involvement

A routine intraoral dental check-up can help detect gum and teeth problems, which are important to monitor over time.

### 3.7. Dermatological Involvement

For skin changes like facial angiofibromas and hypomelanotic macules, dermatoscopy and a Wood’s lamp examination are helpful tools. These are non-invasive tests that can easily reveal these characteristic skin features of TSC.

## 4. Genetic Diagnosis

Tuberous sclerosis complex (TSC) results from mutations in the TSC1 and TSC2 genes. The TSC1 gene (located on 9q34) encodes a protein called hamartin, while the TSC2 gene (located on 16p13.3) encodes tuberin. The TSC2 gene is in close proximity to the PKD2 gene on chromosome 16, which causes the autosomal dominant form of polycystic kidney disease, potentially leading to a contiguous gene deletion syndrome. This results in a complex clinical presentation, which may include severe renal involvement, such as polycystic kidney disease, infantile severe, with tuberous sclerosis (MIM #600273) [44,45,46].

**Figure 2:**
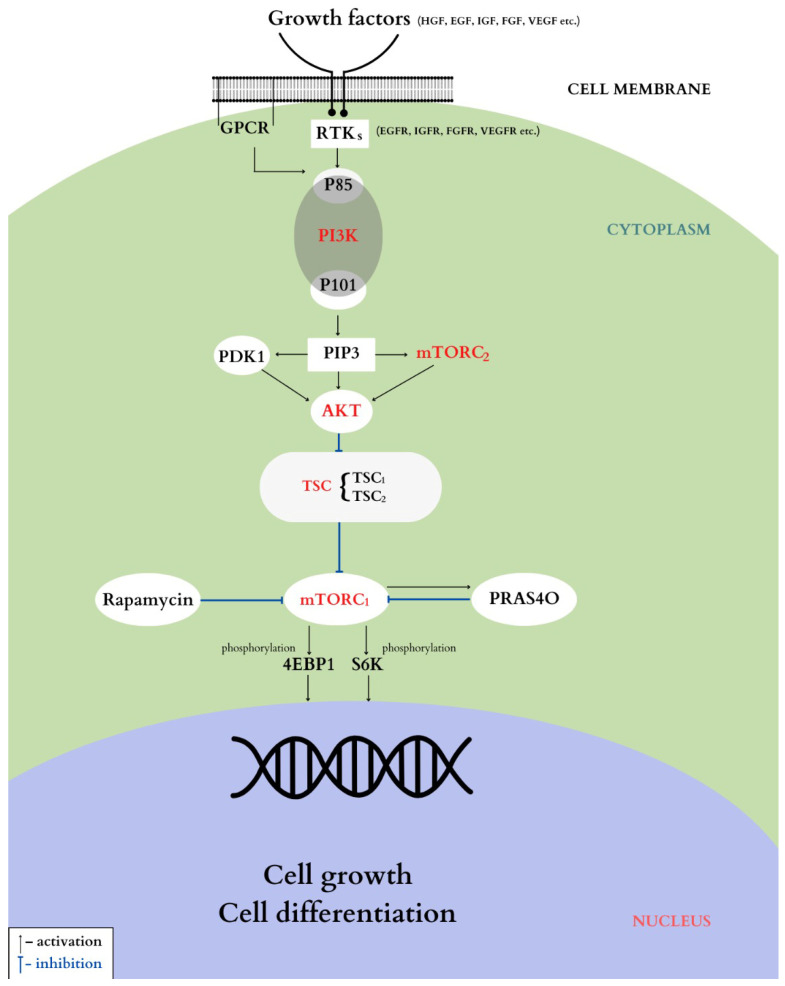
PI3K/AKT/mTOR pathway. Used with the permission of Jurca MC (https://pubmed.ncbi.nlm.nih.gov/36833359/ accessed on 21 December 2024).

The two proteins encoded by the TSC1 and TSC2 genes play a main role in the mTOR signaling pathway, acting as inhibitors of the signaling cascade. The mTOR pathway, interconnected with the PI3K/AKT pathway, is essential for regulating cellular growth, metabolism, and cell survival. Figure 2, shown below, illustrates the mTOR pathway and its interaction with the TSC1 and TSC2 proteins, highlighting their role in cellular processes. The mTOR pathway consists of two major complexes: mTORC1, involved in cellular growth, protein synthesis, and energy metabolism, and mTORC2, which regulates the cellular cytoskeleton, influencing cell shape and movement [47].

Genetic variants in the TSC1 or TSC2 genes disrupt this pathway, leading to the uncontrolled activation of mTOR signaling, which results in excessive cellular division and proliferation. This abnormal proliferation enhances angiogenesis, explaining the development of cutaneous and renal angiofibromas and renal angiomyolipomas. TSC can be inherited or caused by a de novo mutation. In approximately two-thirds of cases, mutations occur de novo, while the remaining third inherit the condition in an autosomal dominant manner [48].

Knudson’s two-hit hypothesis suggests that the disease develops when a mutation in a previously functional allele of TSC1 or TSC2 is combined with a germline mutation in the second allele. Patients with mosaicism often experience a milder clinical presentation, though they are at risk of developing any manifestation of TSC and passing the condition to offspring. Genetic counseling and monitoring are therefore essential for these patients. TSC2 mutations tend to be sporadic and generally present a more severe clinical picture. In familial forms of TSC, the mutational ratio between TSC1 and TSC2 is typically 1:1 [49].

The most common mutations in TSC1 are nonsense and frameshift mutations, leading to the production of a truncated protein, affecting approximately 10–20% of clinically diagnosed patients. TSC2 mutations typically include missense mutations, deletions, or rearrangements, and are found in approximately 70–90% of patients [50,51]. However, molecular diagnosis is negative in 10–25% of patients, due to mutations in introns, the promoter region, or somatic mosaicism [52,53]. Therefore, a negative molecular diagnosis does not rule out TSC.

The pathogenicity of genetic variants in TSC1 or TSC2 is assessed according to the standards of the American College of Medical Genetics (ACMG) for variant interpretation. Pathogenic variants (class 5) clearly disrupt protein synthesis or function, such as nonsense or frameshift mutations and large genomic deletions. Variants whose effect is less certain are not classified as pathogenic unless further evidence supports their role [53]. Advances in genetic testing continue to uncover new pathogenic variants in TSC1 or TSC2. If a pathogenic variant is identified in an affected relative, targeted testing in family members is highly predictive [54,55,56,57].

## 5. Differential Diagnosis

The differential diagnosis of tuberous sclerosis complex (TSC) involves several other conditions, owing to the multi-organ involvement characteristic of TSC. These conditions include the following:

Neurofibromatosis Type 1 (NF-1): This is a monogenic disorder with autosomal dominant inheritance, characterized by cutaneous neurofibromas, café-au-lait spots, and other dermatological manifestations. Tumors may also affect various organs. Molecular diagnosis reveals pathogenic variants in the NF1 gene [58].

Sturge–Weber Syndrome: This condition is marked by a vascular malformation in the brain, which can lead to seizures and neurological disorders. Cutaneous signs include a violaceous port-wine stain (nevus flammeus), typically located on the face [59].

Autoimmune Lymphoproliferative Syndrome (ALPS): this is an immunological disorder characterized by persistent lymphadenopathy, splenomegaly, and other autoimmune manifestations [60].

Overgrowth Syndromes: genetic conditions such as Beckwith–Wiedemann syndrome or Proteus syndrome are associated with excessive tissue proliferation, which may mimic the cutaneous manifestations of tuberous sclerosis [61].

Arteriovenous Malformations (AVMs) and Other Brain Lesions: brain lesions such as AVMs can present with symptoms similar to those of TSC, including seizures or focal neurological deficits [62].

## 6. Treatment

There is no etiologic treatment for tuberous sclerosis complex (TSC). Given the multisystemic involvement of the disease, each affected organ must be evaluated and managed based on the clinical context. A comprehensive multidisciplinary team, including a pediatrician, neurologist, dermatologist, cardiologist, ophthalmologist, psychiatrist, dentist, and other healthcare professionals, is essential for optimal care. Genetic counseling is also beneficial for affected individuals and their families, providing guidance on inheritance patterns and management strategies [63,64].

### 6.1. Use of mTOR Inhibitors (mTORis)

mTOR inhibitors have become the standard treatment for tumor-related manifestations of TSC. The TSC1 and TSC2 genes regulate the mTOR signaling pathway, which is critical for integrating growth signals and transmitting them downstream to mTOR. This pathway’s dysregulation leads to the pathogenesis of TSC, and mTOR inhibitors, such as sirolimus (rapamycin) and everolimus, are used to target these abnormalities. Sirolimus, a macrolide first discovered in 1965 in Streptomyces hygroscopius on Easter Island (Rapa Nui), has demonstrated both immunosuppressive and antineoplastic properties, with its effectiveness in TSC management being established in 2006 [65,66,67,68]. Everolimus, a derivative of sirolimus, has a similar molecular mechanism but differing clinical profiles.

Sirolimus was first approved by the European Medicines Agency (EMA) and the U.S. Food and Drug Administration (FDA) for the treatment of lymphangioleiomyomatosis (LAM) associated with TSC and for preventing organ rejection in transplant patients. It has also been approved for use in treating facial angiofibromas in patients with TSC. Sirolimus has been shown to act more quickly and effectively on facial cutaneous angiofibromas due to its anti-angioproliferative effect, particularly on highly vascularized tumors like those on the face, although it is less effective for nail fibromas and subependymal astrocytomas. The discontinuation of sirolimus therapy often leads to tumor recurrence, although no rebound tumor growth has been observed following treatment cessation [38,69].

In 2020, everolimus was approved by the FDA for the treatment of inoperable subependymal giant cell astrocytomas (SEGAs) in patients with TSC. It is now approved in many countries for treating renal angiomyolipomas and partial-onset epilepsy associated with TSC, in addition to SEGAs [40,70].

Common adverse effects associated with mTOR inhibitors include oral ulceration, hyperlipidemia, and hyperglycemia, which may require dose adjustments or temporary discontinuation of treatment. Clinical trials have reported hyperglycemia in 13% to 50% of patients with cancer treated with mTOR inhibitors. Additionally, sirolimus treatment has been linked to the development of dyslipidemia in transplant patients [71,72,73,74,75].

### 6.2. Metformin Treatment

Metformin, an oral antidiabetic agent, plays a crucial role in regulating the mTOR signaling pathway by activating the serine/threonine kinase AMPK. When cellular energy levels are low, metformin inhibits mitochondrial complex I, activating AMPK, which senses the energy status of the cell and suppresses cell growth when energy levels are reduced [47,76,77]. To maintain energy balance, AMPK phosphorylates various downstream molecules, promoting catabolic processes and inhibiting ATP-consuming anabolic pathways [78]. However, the precise molecular mechanisms underlying its therapeutic role are still not fully understood.

Metformin inhibits the mTORC1 complex through several mechanisms, including the following:

(a) The activation of the TSC1 and TSC2 genes via AMPK, which inhibits mTORC1;
(b) The direct phosphorylation of raptor, a key regulator of mTORC1, by AMPK, leading to mTORC1 inhibition;
(c) Blocking IGF-1 and insulin signaling, which further inhibits mTORC1 activity through the TSC;
(d) The induction of the p53 protein, which also inhibits mTORC1;
(e) Increasing the expression of the DICER1 gene, involved in RNA and pre-microRNA breakdown, with mutations linked to tumor syndromes;
(f) The suppression of hypoxia-inducible factor (HIF-1α), which regulates the cellular response to hypoxia through AMPK and mTORC1;
(g) The inhibition of fatty acid synthase, the enzyme responsible for fatty acid production;
(h) The direct inactivation of Ragulator, disrupting its activation of mTORC1 via Rheb.

Each of these mechanisms contributes to the overall inhibitory effect of metformin on mTORC1, which is crucial for regulating cell growth and metabolism [79]. As an anticancer agent, metformin also inhibits the proto-oncogene c-MYC, which is overexpressed in several tumor types [80].

**Figure 3:**
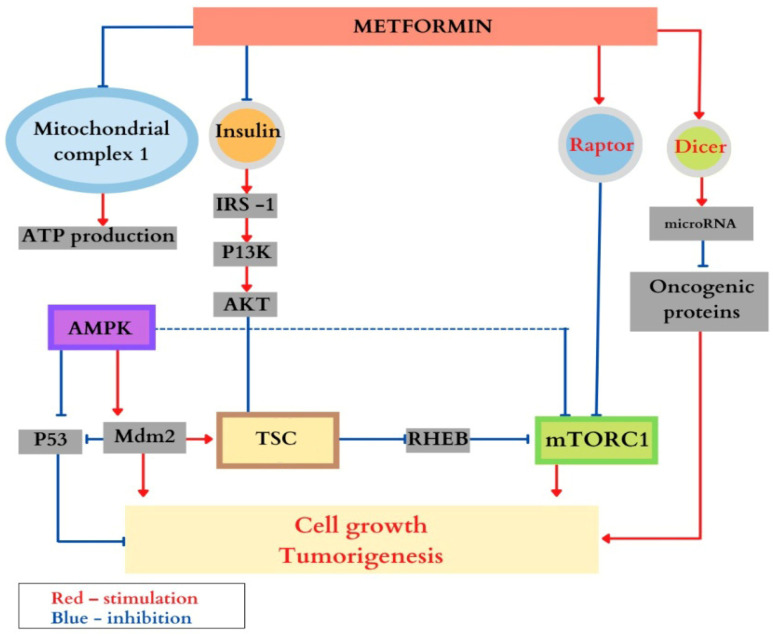
The most important effects of metformin are summarized in this figure. Used with permission from Jurca MC [https://pubmed.ncbi.nlm.nih.gov/36833359/ accessed on 22 December 2024].

Figure 3 summarizes the main effects of metformin in the context of its therapeutic role in tuberous sclerosis complex (TSC).

The primary effects of metformin are summarized in Figure 3. However, there are currently a limited number of studies on the effectiveness of metformin in TSC. One randomized multicenter study by Amin et al. followed patients with TSC, comparing the effects of metformin to a placebo on renal angiomyolipomas, subependymal astrocytomas, and epileptic seizures. The results showed significant volume reduction in both renal angiomyolipomas and subependymal astrocytomas, and a decrease in the frequency of epileptic seizures, demonstrating that metformin is a safe and well-tolerated treatment, both in the pediatric population and in adults with TSC [47,81].

## 7. Surveillance, Quality of Life, and Care Burden

Tuberous sclerosis complex (TSC) can impose a severe physical, psychological, and financial burden on both individuals with TSC and their caregivers. This burden is often compounded by neuropsychiatric conditions that many patients experience. Managing the stress associated with TSC and its complexities is crucial for the mental well-being of both the individual with TSC and their caregivers. Healthcare providers should support families by connecting them with secondary care teams, including therapists, social workers, and counselors. Access to regional resources such as respite care and mental health support programs is essential. Additionally, the physical and mental health of caregivers and siblings should not be overlooked.

The TSC International Consensus Group, composed of over 80 specialists from 14 countries with diverse medical expertise, has issued updated consensus recommendations. These recommendations are based on extensive practical experience and the best available scientific evidence, providing the most up-to-date standard of care for individuals with TSC throughout their lifetime, regardless of location. However, significant barriers at the individual, regional, or national levels may limit access to certain technologies, treatments, or medical specialists needed for some of these consensus recommendations. Developmental and behavioral screening should be performed every 6–12 months in infancy and early childhood and as needed later; skin and dental surveillance is recommended annually; kidney surveillance every 1–3 years; neurological and neuroimaging monitoring every 1–3 years before the age of 25 and as needed after; cardiac assessment annually before the age of 3 and every 3–5 years after; and high-resolution CT of lungs in adult females every 5–10 years.

Local and international support groups play a critical role in helping individuals and families navigate the TSC care landscape, offering psychosocial support and educating healthcare providers as well as those affected by TSC [82,83].

## 8. Care Coordination and Transition from Pediatric to Adult Care

The transition from pediatric to adult healthcare is particularly challenging for individuals with chronic diseases, such as tuberous sclerosis complex (TSC), due to the involvement of multiple organ systems requiring specialized care. Ideally, a multidisciplinary team should manage this clinical transition to ensure comprehensive care. The Child Neurology Foundation recently convened a multidisciplinary team that produced a consensus statement outlining eight key principles defining the role of pediatric neurologists in this critical process.

Given the complexity and variety of TSC manifestations over the lifespan, support from local healthcare professionals (e.g., primary care physicians) is essential in addition to coordinated care from TSC specialists and other healthcare providers. These specialists are often concentrated in regional or national referral centers, which can complicate access to care for some individuals.

In countries without established government policies for the support of patients with rare diseases, research initiatives supported by patient advocacy groups—often centered around university hospitals—have played a crucial role in establishing multidisciplinary medical teams to care for individuals with TSC. These initiatives help bridge gaps in care, ensuring continuity and comprehensive management for patients as they transition into adulthood [84,85].

### New Trends in the EU

In recent years, there have been several important trends in the European Union [86,87] regarding care coordination and transition for patients with TSC:

EU Rare Disease Strategy and Plans—The European Commission’s Rare Disease Strategy, launched under the EU’s Health Program, aims to provide better coordination of care and treatment for individuals with rare diseases, including TSC. This strategy promotes the establishment of European Reference Networks (ERNs) for rare diseases, ensuring that specialized care is available across borders. This network helps improve access to expertise and ensures continuity of care as patients transition from pediatric to adult healthcare services.

Cross-Border Healthcare—the EU’s Cross-Border Healthcare Directive facilitates access to healthcare services in other EU member states, enabling patients with TSC to seek care in centers with specialized expertise even if they are located outside their home country. This has been particularly valuable for individuals transitioning from pediatric care, where specialized adult services may not be available locally.

Patient-Centered Care Models—There is a growing shift in the EU toward patient-centered care models that emphasize personalized treatment plans and the active involvement of patients and their families in care decisions. In the context of TSC, this trend supports tailored care pathways that account for the unique needs of individuals as they age and face new challenges in adult life.

Integration of Digital Health and Telemedicine—The use of digital health tools and telemedicine is increasing across the EU, especially following the COVID-19 pandemic. These tools are enhancing access to care for individuals with TSC, particularly in rural or underserved areas. Telemedicine enables consultations with specialists in other regions, ensuring that patients continue to receive expert advice even when transitioning to adult care.

Genetic Counseling and Long-Term Support—As genetic testing and counseling for rare diseases, including TSC, become more widespread in the EU, there is a growing emphasis on providing long-term support for families. Genetic counseling services are being integrated into patient care, helping individuals and families navigate not only the medical aspects of TSC but also the social, emotional, and psychological impacts. This is particularly important as individuals with TSC transition from pediatric to adult care, where life planning and family dynamics may change.

Policy and Funding Support for Rare Diseases—In several EU countries, there has been an increase in policy and funding support for rare diseases, including TSC. These efforts are aimed at improving access to care, encouraging research, and ensuring that the unique needs of patients with rare diseases are addressed. As part of these efforts, there is a growing focus on ensuring that the transition to adult care is seamless, with continued support for patients throughout their lives.

## 9. Conclusions

Tuberous sclerosis complex (TSC) is a complex genetic disorder with significant clinical variability, greatly impacting patients’ quality of life. The discovery of the TSC1 and TSC2 genes, along with the dysregulation of the mTOR pathway, has provided crucial insights into the molecular mechanisms of the disease and has paved the way for the development of targeted therapies. mTOR inhibitors, particularly everolimus, have proven to be effective in controlling tumor growth and alleviating neurological and neuropsychiatric symptoms, offering considerable improvements in patient outcomes.

Given the multifaceted nature of TSC, personalized and multidisciplinary approaches remain vital for optimizing patient care. These approaches should focus on addressing the specific clinical needs of each patient, considering the variability of manifestations. Moreover, future research efforts should aim at developing novel therapies, enhancing the effectiveness of current treatments, and further exploring the factors that contribute to clinical variability in TSC. Such advancements will improve both management and quality of life for individuals affected by this rare genetic disorder.

## Figures and Tables

**Table 1:** Diagnostic criteria for TSC.

Major Disease Criteria	Minor Disease Criteria
**1.** **Depigmented macules with a diameter of 3–5 mm** **2.** **Facial angiofibromas (more than 3)** **3.** **Ungual fibromas (more than 2)** **4.** **“Shagreen” patch** **5.** **Retinal hamartomas** **6.** **Cortical dysplasia** **7.** **Astrocytomas** **8.** **Subependymal nodules** **9.** **Cardiac rhabdomyomas** **10.** **Lymphangioleiomyomatosis** **11.** **Renal angiomyolipomas**	Intraoral fibromas Dental enamel abnormalities Retinal white spots Renal cysts Other hamartomas Sclerotic bone changes “Confetti” skin lesions
**Observations:** **-** **Definite TSC: 2 major features or 1 major feature with 2 minor features.** **-** **Probable TSC: 1 major criterion and 1 minor criterion.** **-** **Possible TSC: either 1 major feature or ≥2 minor features [8].**	

## Data Availability

Not applicable.
